# *Healthy from the Start*: Co‐Designing Sleep, Nutrition and Physical Activity Resources for Young Shiftworkers—Novel Implementation and Evaluation

**DOI:** 10.1111/hex.70063

**Published:** 2024-10-15

**Authors:** Alexandra E. Shriane, Sally A. Ferguson, Gabrielle Rigney, Charlotte C. Gupta, Tracy Kolbe‐Alexander, Madeline Sprajcer, Cassie Hilditch, Robert Stanton, Matthew J. W. Thomas, Jessica L. Paterson, Jamie Marino, Grace E. Vincent

**Affiliations:** ^1^ Appleton Institute CQUniversity Wayville South Australia Australia; ^2^ School of Health and Medical Sciences, Faculty of Health, Engineering and Sciences University of Southern Queensland Ipswich Queensland Australia; ^3^ Centre for Health Research University of Southern Queensland Springfield Queensland Australia; ^4^ Division of Exercise Science and Sports Medicine, Department of Human Biology, Faculty of Health Sciences University of Cape Town Cape Town South Africa; ^5^ Fatigue Countermeasures Laboratory San Jose State University San Jose California USA; ^6^ Cluster for Resilience and Wellbeing, Appleton Institute CQUniversity Wayville South Australia Australia; ^7^ Flinders Institute for Mental Health and Wellbeing Flinders University Adelaide South Australia Australia

**Keywords:** health information, health resources, mixed methods, non‐standard work, participatory, young adult

## Abstract

**Introduction:**

The increasing prevalence of shiftwork among young adults poses significant health risks, primarily due to its disruptive effects on sleep, nutrition and physical activity. Addressing these risks necessitates the development of tailored, evidence‐based resources to support these key health behaviours. Participatory research approaches, engaging those with relevant lived experience (i.e., co‐design) are a novel and effective approach in developing these resources. As such, the aim of the present study was to explore whether sleep, nutrition and physical activity resources for young shiftworkers could be developed using participatory, co‐design approaches and how co‐designers would rate both the approaches used and the resulting resources.

**Methods:**

A participatory approach engaged co‐designers (young, experienced or previous shiftworkers; workplace health and safety specialists; science communicators and academic experts) to complete 2–3 online questionnaires and participate in 1–2 online workshops, to co‐design sleep, nutrition and physical activity resources for young shiftworkers. Following resource development, co‐designers assessed both the participatory approach and the resulting resources, through an online questionnaire, which included the Public and Patient Engagement Evaluation Tool (PPEET).

**Results:**

Co‐designers (*n* = 48) participated in the development of sleep, nutrition and physical activity resources for young shiftworkers. Co‐designers evaluated the participatory approach positively, with a mean rating across all PPEET items of 4.7 (±0.2) on a 5‐point Likert scale. Co‐designers also provided positive ratings for the resources, with the majority (91.7%) either *agreeing* or *strongly agreeing* that they were user‐friendly, valuable and informative for young shiftworkers and would serve as a credible source of health information.

**Conclusion:**

By adopting a novel participatory approach, we successfully co‐designed sleep, nutrition and physical activity resources for young shiftworkers. Participatory approaches, including co‐design, should be considered when developing health interventions for shiftworkers, given the value of embedding lived experience to address their unique lifestyle challenges.

**Patient or Public Contribution:**

Co‐designers and/or people with relevant lived experience were involved in all project activities: conceptualisation, design, recruitment, data collection, data analysis, knowledge translation and output generation.

## 1 Introduction

Shiftwork is a cornerstone of modern life, involving employment that falls outside of conventional daytime hours to include early morning, evening and night work [1, 2, 3]. A wide range of industries, such as healthcare, transport and manufacturing, rely on shiftwork to deliver around‐the‐clock services [1]. While shiftwork has important economic and societal benefits [2], it is also linked with a range of adverse health and wellbeing outcomes [3], including overweight and obesity [4], diabetes [5], cancer [6], mental health problems [7], cognitive impairment [8] and social isolation [9], as well as accidents, injuries and errors in the workplace [10]. These problems often develop as a consequence of the challenges shiftworkers experience in maintaining healthy lifestyle behaviours, including obtaining sufficient quality sleep [3], consuming a nutritious diet [11] and engaging in adequate amounts of physical activity [12].

In managing their health and wellbeing, while balancing the demands of employment outside traditional working hours, shiftworkers may seek out information on optimising their sleep, nutrition or physical activity [13, 14]. Additionally, organisations that employ shiftworkers may offer information or education on healthy behaviours [15, 16]. While there is an abundance of information on sleep, nutrition and physical activity available to the general population, it is rarely tailored for the unique demands of shiftwork. For example, sleep hygiene advice available to the general population suggests limiting or altogether avoiding napping during daytime hours, contradicting fatigue management advice for shiftworkers, which recommends napping as an effective strategy to decrease fatigue and improve waketime performance [17, 18]. As such, the development of tailored, evidence‐based information on sleep, nutrition and physical activity is urgently needed.

While providing shiftworkers with tailored, evidence‐based information on healthy behaviours is important, when (e.g., at the commencement of shiftwork, after a fatigue‐related incident) and how (e.g., workplace education, publicly available resources) this information is delivered are critical considerations. In countries like Australia, the United States of America and the United Kingdom, approximately 15%–20% of working adults are shiftworkers [19, 20]. While the overall prevalence of shiftwork has remained stable over the past few decades, the proportion of young adults (18–25 years old) participating in shiftwork has been steadily increasing [21, 22]. As such, providing young adults with information on their sleep, nutrition and physical activity as they enter shiftwork may be an important point of intervention, particularly given the link between engaging in shiftwork in young adulthood and poor health outcomes by middle age [22]. This early intervention approach is efficacious and widely accepted across the public health sector, with individuals demonstrating better long‐term health and wellbeing outcomes when information, education and health promotion efforts are implemented during young adulthood [23, 24]. Further, while the long‐term impacts of shiftwork are well‐documented, there is evidence suggesting that the commencement of, and even short‐term (1–2 years) engagement in, shiftwork carries its own negative impacts on health and wellbeing in the immediate term [25]. For example, sleep and mental health outcomes are significantly impacted soon after the commencement of shiftwork, including a decline in sleep duration, increases in depressive symptoms and signs of burnout, suggesting that negative health outcomes may be apparent much earlier than previously documented [25].

Recent evidence suggests that shiftworkers will engage more readily with information that is tailored to their unique lifestyles and work commitments, than with generic health and wellbeing advice [26]. To achieve the desired nuance in tailoring health and wellbeing information for young shiftworkers, input from young shiftworkers themselves, as well as other key stakeholders with lived experience in shiftworker health and wellbeing should be sought. More specifically, this should include lived experience co‐designers, experts by experience and/or citizen scientists [27, 28]. The meaningful involvement of these experts can be achieved through experience‐based co‐design (co‐design) [28]. Co‐design projects have been used in health and wellbeing contexts since the late 20th century and continue to gain traction for their ability to embed the knowledge and experience of end‐users into research processes and outcome translation [29]. In turn, co‐designed outputs often demonstrate greater applicability and acceptability to end‐users than those produced solely through traditional scientific paradigms [29]—something that is crucial in overcoming challenges of shiftworker engagement with non‐tailored health information [27].

Sleep, nutrition and physical activity resources for the general population have all benefited from development through co‐design projects [30, 31, 32, 33, 34]; however, to date, such approaches have had limited implementation in a shiftwork context. Instead, most co‐design projects related to shiftwork have focused on occupational factors or rostering practices [35, 36], rather than the health and wellbeing of the individual worker. For the purposes of this project, the use of co‐design is conceptualised in a broader Participatory Action Research (PAR) approach. A central tenet of PAR is the understanding that those who are most impacted by the outcomes of the research (i.e., end‐users) are situated within the research itself [37]. In PAR, end‐users are seen as equal partners in information gathering and meaning making, rather than simply being the subjects of data collection [38]. In addition to the participatory nature of the approach, as achieved through co‐design, elements of reflexive action must also be incorporated, demonstrating a purposeful change in the way that things are done or seen as a result of the project [39]. Feedback mechanisms should be embedded in the project to allow for reflection on both the methodology used and the resources developed, allowing for learning from participant experiences and reflections for the benefit of future investigations. Therefore, to address the paucity of tailored, co‐designed health behaviour interventions designed with, and for, young shiftworkers, we co‐designed *Healthy from the Start*, a freely available early intervention suite of resources designed to optimise young shiftworkers' sleep, nutrition and physical activity.

### 1.1 Aims

The overarching purpose of the *Healthy from the Start* project was to co‐design sleep, nutrition and physical activity resources for young shiftworkers. However, given the novelty of using a co‐design approach in a shiftwork context, this paper aims to explore the implementation and evaluation of this participatory, co‐designed approach, separate to the resulting resources. The following research questions will be explored:

1. Did co‐designers (lived experience co‐designers and academic experts) effectively participate in activities to successfully produce sleep, nutrition and physical activity resources for young shiftworkers?
2. What were the views of participants (lived experience co‐designers) on the quality and utility of sleep, nutrition and physical activity resources for young shiftworkers, as developed in this project?
3. What were the views of participants (lived experience co‐designers) on the participatory, co‐design approaches used in developing sleep, nutrition and physical activity resources for young shiftworkers?

## 2 Methods

### 2.1 Design

**Figure 1:**
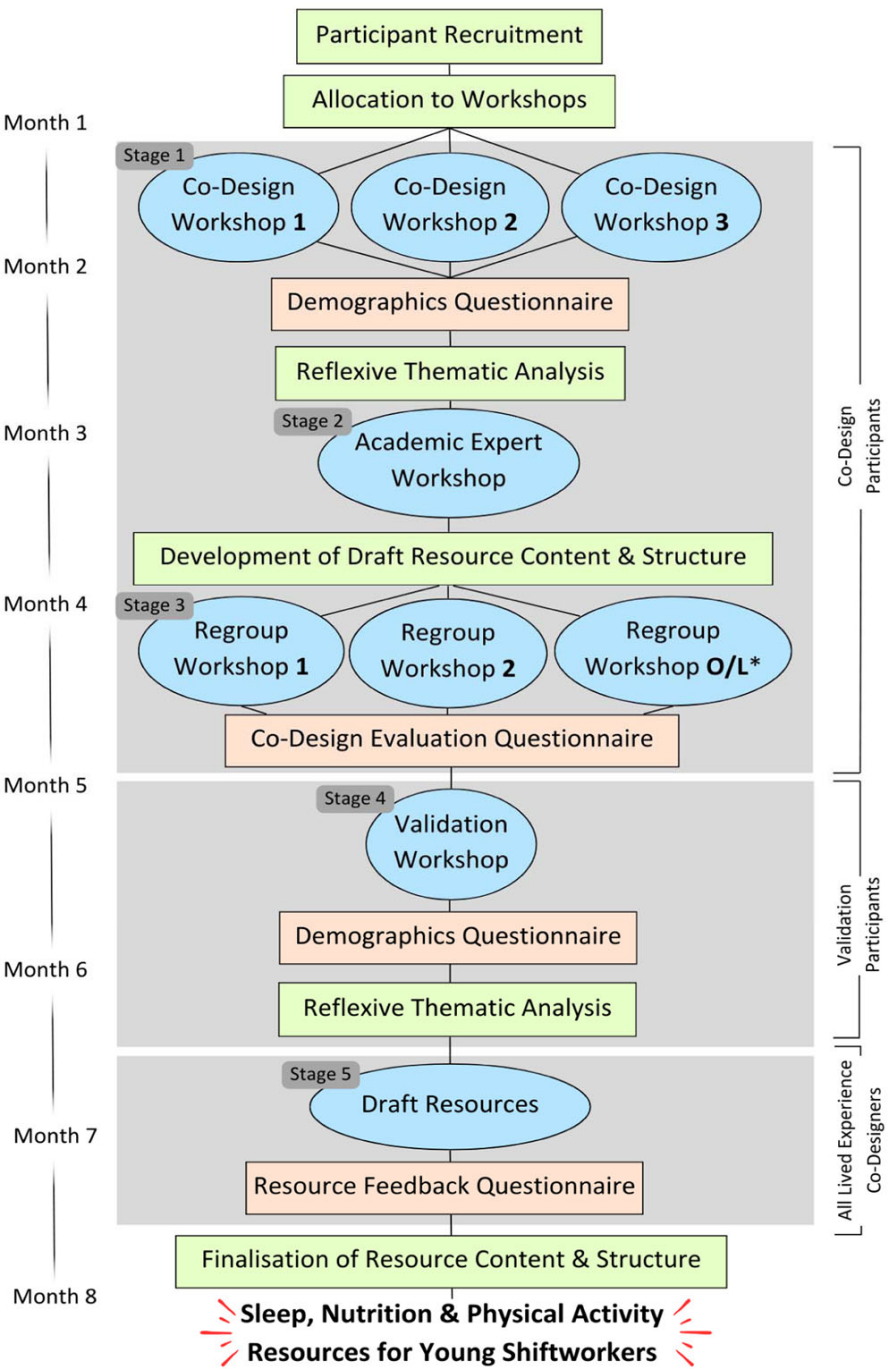
*Healthy from the Start* co‐design stages. *O/L denotes offline workshop participation.

A participatory, co‐design approach was used, applying methodological flexibility to suit the population, project and problem [39, 40, 41]. As illustrated in Figure 1, this was achieved through ongoing, iterative engagement with co‐designers comprising young shiftworkers, experienced shiftworkers, previous shiftworkers, workplace health and safety experts, science communications specialists and academic experts in shiftworker health and wellbeing. Additionally, the project lead (A.E.S.) was a long‐term healthcare shiftworker, and members of the project team have conducted overnight laboratory studies for many years, ensuring that the project was conceptualised and conducted through the lens of people with lived experience of shiftwork.

Co‐designers engaged in a series of workshops and online questionnaires to facilitate mixed methods data collection, with data informing the development of a suite of sleep, nutrition and physical activity resources for young shiftworkers. Crucially, process and outcome evaluative components were embedded in the latter stages of the project, facilitating learning in relation to the use of a participatory, co‐design approach in this context and the quality of the subsequent resources developed [41].

Ethics approval was provided by the Human Research Ethics Committee at Central Queensland University, Australia, before commencing participant recruitment (Approval No. 24460).

### 2.2 Participants

Table 1 outlines the characteristics and eligibility criteria of each participant group, which together, formed the co‐design group for the project. Participants with lived experience relevant to young shiftworker health and wellbeing included: young shiftworkers, experienced shiftworkers, previous shiftworkers and workplace health and safety experts. To ensure effective communication of information within the resources, science communications specialists with experience in young adult communication were also recruited. Finally, to ensure that the resources were concordant with the existing evidence base on health and wellbeing, academics with expertise in sleep, nutrition and physical activity literature in relation to shiftwork formed the broader project team.

**Table 1:** Eligibility criteria for co‐designers.

Participant category	Eligibility criteria^a^
Young shiftworkers	Aged 18–25 years Engaged in full‐time shiftwork employment.
Experienced shiftworkers	Engaged in full‐time shiftwork employment for ≥ 5 years.
Previous shiftworkers	Were engaged in full‐time shiftwork for ≥ 5 years Ceased shiftwork within the last 5 years.
Workplace health and safety experts	Currently employed in a role where they provide advice to/oversight of shiftworker health and wellbeing Have prior experience in providing advice to/oversight of shiftworker health and wellbeing.
Science communications specialists	Currently employed in a role where they are responsible for communicating health and/or science information with young adults Have prior experience in communicating health and/or science information with young adults.
Academic experts (project team)	Demonstrable expertise in the evidence base on shiftwork in relation to: ○ Sleep ○ Nutrition ○ Physical activity.

^a^ All lived experience participants (i.e., those not part of the project team) were asked to confirm that they were located in Australia, and had access to an internet‐connected device to participate in workshops and complete online questionnaires.

Between 40 and 50 co‐designers (i.e., both lived experience co‐designers and academic experts) were sought, encompassing all categories as described in Table 1, with shiftworkers (young, experienced and previous) purposefully recruited to form the majority of participants, with young shiftworkers the largest cohort within this group. This was deemed an appropriate number to populate sufficient workshops to achieve data saturation, while allowing for potential attrition [42].

### 2.3 Recruitment

Participants were recruited through two channels: social media posts and existing professional networks. Existing professional networks comprised individuals who met the inclusion criteria, as described in Tables 1, 2 and 2, and were known to the project team in a professional context. As such, this network spanned a range of industries and professions (e.g., mining, manufacturing, transport, healthcare and emergency services). To ensure diversity of experience and perspective amongst participants, social media posts were also used to reach a broad community sample.

Recruitment material provided a brief overview of the project and directed interested individuals to an online landing page, where a participant information sheet and enrolment questionnaire were available. To enrol and provide informed consent, all participants were asked to confirm that they met eligibility criteria, as described in Table 1, with participants able to self‐select the category they felt most equipped to represent, where necessary (i.e., some participants may have met eligibility criteria for more than one category; e.g., a workplace health and safety expert who previously engaged in shiftwork). During enrolment, participants were informed that they would be remunerated with an AUD150.00 electronic giftcard for each relevant stage they completed (described below) and that they were encouraged to discuss the aims of the project, and their participation in it, with relevant stakeholders (e.g., other shiftworkers, workplace health and safety experts) to allow for a diversity of opinions during data collection.

### 2.4 Procedure

A combination of workshops and online questionnaires were utilised to facilitate mixed methods data collection, blending both digital face‐to‐face (i.e., workshops; as part of a group) and digital asynchronous (questionnaires; as an individual) involvement, which allowed for maximum participation [42, 43]. Workshops were held online using Zoom (2024; Version 5.17.11), while questionnaires were completed online through Qualtrics (2024; Provo, Utah, USA). All workshops ran for approximately 60 min, were audio and video recorded (with participants requested to have their cameras on if they felt comfortable doing so) and facilitated by the project lead (A.E.S.) with support from additional team members (G.E.V., C.C.G., J.M.). Participants were encouraged to contribute verbally during the workshops, however, were also able to contribute via the meeting chat, which was included in the transcription after each workshop.

To investigate participant feedback on the developed resources, online questionnaires presented a combination of Likert‐type scale ratings and free‐text response questions. Likert‐type scale questions aimed to understand how lived experience co‐designers rated the quality, functionality, value and credibility of the developed resources, while free‐text questions aimed to capture what emotions lived experience co‐designers believed young shiftworkers may feel, or the action they may take, in response to engaging with the resources. To understand participant experiences throughout the project, and to evaluate the participatory, co‐design approach chosen, lived experience co‐designers were asked to complete the Public and Patient Engagement Evaluation Tool (PPEET) [44]. This 14‐item tool asks participants to rate their agreement with a range of statements using a 5‐point Likert‐type scale (from 1 *strongly disagree* to 5 *strongly agree*) on participation elements, such as the ability to express their views freely, receiving support to participate and participation being a good use of their time. For each item, a higher score is interpreted as a more positive engagement experience. Three additional items were added by the project team, two of which were answered on the same 5‐point Likert‐type scale as the PPEET—one to understand how relevant the types of recruited co‐designers were to the project outcomes, and one to understand whether remuneration was appropriate in relation to participation time and effort. The third item added by the project team asked co‐designers to rate what percentage of their contributions (out of 100) they felt had been considered and included in the developed resources. Higher scores for each of these additional items were interpreted as more positive engagement.

#### 2.4.1 Stage 1: Co‐design Workshops—Content Code and Theme Development

Upon enrolment, co‐designers were allocated to one of three Co‐Design Workshops. In allocating co‐designers, a consistent mix of participation category (i.e., young shiftworker, experienced shiftworker, previous shiftworker, workplace health and safety expert, science communications specialist) was sought. This was done purposefully to ensure that there was a similar level of diversity across workshops (i.e., roughly the same number of each group at each workshop), with workshops averaging nine co‐designers and attended by four facilitators. Limiting the number of academic experts in the workshops elevated the voices of lived experience co‐designers through a facilitated discussion on what they believed young shiftworkers needed to know about sleep, nutrition and physical activity (see Supporting Information S1: File A). Questions to commence or prompt discussion were posed by the facilitator; however, the discussion itself occurred amongst co‐designers, with limited input from the facilitator or other academic experts in attendance. This approach allowed for free‐flowing conversation and sharing of ideas in relation to young shiftworker health and wellbeing, without the ‘sway’ of academic opinion or the evidence base. A recording of each Co‐Design Workshop was provided to the broader project team to review after completion, to inform their participation in Stage 2.

At the conclusion of each Co‐Design Workshop, lived experience co‐designers were asked to complete a brief online questionnaire, which captured demographic information, including age, gender, occupation, industry and years of experience. Co‐Design Workshops were then transcribed verbatim, as per Braun and Clarke's approach to reflexive thematic analysis [45, 46], to elucidate unique codes relating to each health behaviour (sleep, nutrition and physical activity). These codes were then grouped into themes, which informed the structure and content of draft resources.

#### 2.4.2 Stage 2: Academic Expert Workshop—Draft Resource Content Development

The codes and themes developed through analysis of the Co‐design Workshop transcripts were presented to the academic expert co‐designers (project team) during a team workshop. The purpose of this workshop was to explore the key messages that lived experience co‐designers believe should be communicated with young shiftworkers, and develop content based on input from academic experts, therefore ensuring alignment with the broader evidence base on shiftworker health and wellbeing. For example, if a code had emerged from the Co‐Design Workshops in relation to caffeine intake, academic expert co‐designers were asked to confirm the specific advice that should be provided based on the literature. Consistent with reflexive thematic analysis, academic experts were also asked to recommend any additional codes or themes that had not emerged during the Co‐Design Workshops, based on their knowledge of the shiftwork evidence base. This workshop resulted in an updated set of codes and themes, as well as drafted resource copy for presentation back to lived experience co‐designers in Stage 3.

#### 2.4.3 Stage 3: Regroup Workshops—Draft Resource Content Refinement

Following the development of draft resource content and structure from Stages 1 and 2, Regroup Workshops were held, in which lived experience co‐designers were brought back together, with the same facilitator (A.E.S.) and team members (G.E.V., C.C.G., J.M.) in attendance. The purpose of these workshops was to ensure that data analysis had resulted in a correct interpretation of the discussions from the Co‐Design Workshops. Further, they confirmed the accurate translation of the information shared by lived experience co‐designers into draft resources via academic expert input. The codes and themes distilled through analysis were presented, along with draft resource document that aligned with each code and/or theme. For each health behaviour (sleep, nutrition and physical activity), lived experience co‐designers were asked to provide verbal and/or written feedback (via meeting chat) on the codes, themes and draft copy (see Supporting Information S1: File B).

At the conclusion of each Regroup Workshop, participants were asked to complete an online questionnaire that used the 14‐item Public and Patient Engagement Evaluation Tool (PPEET) [45] to evaluate the co‐design approach used for the project, in addition to the three items added by the project team (on relevance of co‐designers, remuneration and input captured). At the end of this questionnaire, participants were asked whether they wished to provide feedback on the developed resources (Stage 5).

Participants who were unable to attend a Regroup Workshop (e.g., shiftworkers whose work commitments conflicted with workshop scheduling) were able to contribute offline; these participants were sent a recording of the facilitator presenting the codes, themes and draft copy and asked to provide their feedback through an online form (as opposed to verbally during the workshop), before completing the same evaluation questionnaire.

#### 2.4.4 Stage 4: Validation Workshop—Draft Resource Content Validation

To pilot the draft resource content and structure before formal creative development, a subgroup of young shiftworkers who had not been involved in the prior stages were recruited for a Validation Workshop. These young shiftworkers were recruited through the same channels as the previous cohort (social media and existing professional networks) and completed the same enrolment processes. A similar approach was used to that of the Regroup Workshops, with young shiftworkers being presented with codes, themes and draft copy for sleep, nutrition and physical activity resources (see Supporting Information S1: File B). For each health behaviour, young shiftworkers were asked to provide verbal and/or written (via workshop chat) feedback on the draft resources, and also identify whether there were any additional codes or themes that should be added. At the conclusion of the Validation Workshop, participants were asked to complete the same demographic questionnaire as other workshop participants, and also asked whether they wished to provide feedback on the resources (Stage 5).

#### 2.4.5 Stage 5: Co‐Designer Feedback—Final Resource Validation

Following the Validation Workshops, the finalised resource structure and content were provided to creatives (graphic designer, animator) for development into a suite of resources: a website, a series of pictorial infographics and an animated video. Further details on the resources, including their content, will be presented in forthcoming publications. Once developed, lived experience co‐designers who had been nominated to provide feedback were emailed online copies of the resources, which were yet to be made publicly available, along with an online questionnaire to capture their feedback. Feedback was reviewed, and the resources further refined by the project team, to result in a final set of sleep, nutrition and physical activity resources for young shiftworkers.

## 3 Results

### 3.1 Research Question 1: Co‐Designer Participation

**Figure 2:**
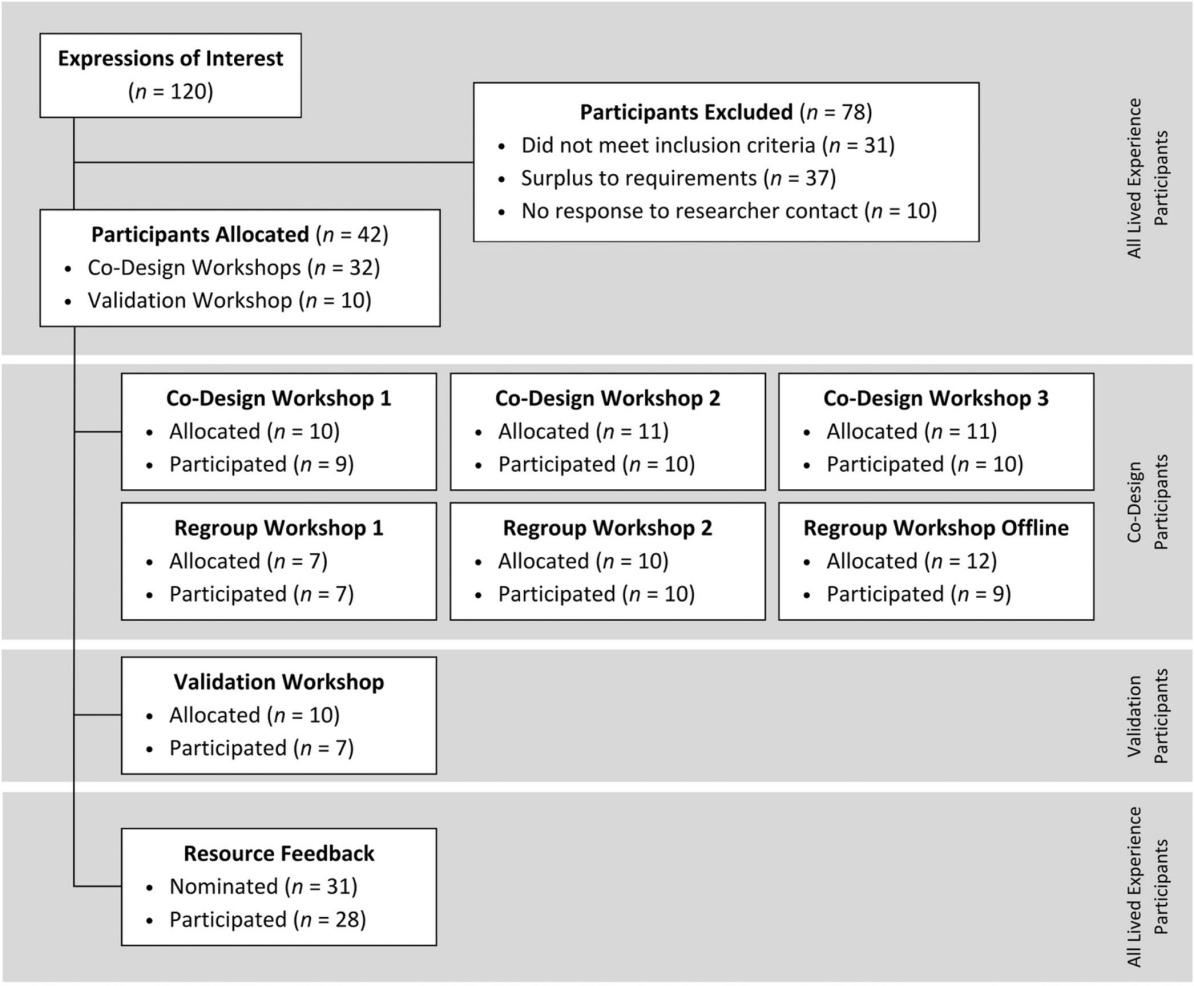
Recruitment and retention of lived experience co‐designers.

Participant inclusion, exclusion and retention are illustrated in Figure 2, while participant demographic information is presented in Table 2 and work characteristics in Table 3. Lived experience co‐designers (i.e., all participants except the academic experts) were recruited between September 2023 and January 2024, with 120 individuals expressing interest in participating. Of these, 42 (35.0%) were eligible for participation and allocated to workshops (see Figure 2 for exclusion conditions), with 36 of these (80.0%) participating in at least one workshop. As per purposive recruitment, the largest group of lived experience co‐designers were young shiftworkers (*n* = 14, 38.9%), followed by experienced shiftworkers (*n* = 9, 25.0%). Across all lived experience co‐designers, the mean age was 35.1 years (±6.44 years), with 22 participants (61.1%) identifying as female. Of those who were eligible to elect to review resources (i.e., those that completed both Co‐Design and Regroup Workshops, or participated in the Validation Workshop; *n* = 33), the majority (*n* = 31, 93.9%) nominated to do so, with most of these (*n* = 28, 90.3%) completing the feedback questionnaire. All academic experts who joined the project (*n* = 12) remained engaged and part of the project team throughout.

**Table 2:** Demographics of lived experience co‐designers.

	Participants (*n* = 36)	Age (mean ± SD)	Gender (*n*, % female)	Years of experience (mean ± SD)
Young shiftworkers	14 (38.9%)	23.9 ± 1.8	11 (78.6%)	2.8 ± 2.0
Experienced shiftworkers	9 (25.0%)	31.7 ± 9.0	3 (33.3%)^a^	15.4 ± 10.20
Previous shiftworkers	3 (8.3%)	40.0 ± 10.2	2 (66.6%)	17.0 ± 10.0^b^
Workplace health and safety experts	7 (19.5%)	47.7 ± 8.3	3 (42.9%)	18.4 ± 9.3
Science communications specialists	3 (8.3%)	32.0 ± 2.9	3 (100%)	9.0 ± 4.2

^a^ *n* = 1 participant preferred not to disclose their gender.

^b^ Years of experience as a shiftworker (i.e., before leaving shiftwork).

**Table 3:** Work characteristics of lived experience co‐designers.

	Participants *n* = 36
Industry (all participants)^a^	
Healthcare and Social Assistance	18 (50.0%)
Professional, Scientific and Technical Services	9 (25.0%)
Mining	7 (19.4%)
Public Administration and Safety	6 (16.7%)
Construction	5 (13.9%)
Education and Training	5 (13.9%)
Information media and Telecommunications	5 (13.9%)
Transport, Postal and Warehousing	5 (13.9%)
Administrative and Support Services	3 (8.3%)
Agriculture, Forestry and Fishing	3 (8.3%)
Electricity, Gas, Water and Waste Services	3 (8.3%)
Manufacturing	3 (8.3%)
Accommodation and Food Services	2 (5.6%)
Financial and Insurance Services	1 (2.8%)

^a^ Participants were able to select all industries in which they had relevant experience.

### 3.2 Research Question 2: Resource Feedback Outcomes

Of the 31 participants nominated to provide feedback on the final suite of resources (encompassing both co‐design and validation phase participants), 28 (90.3%) completed the feedback questionnaire.

**Figure 3:**
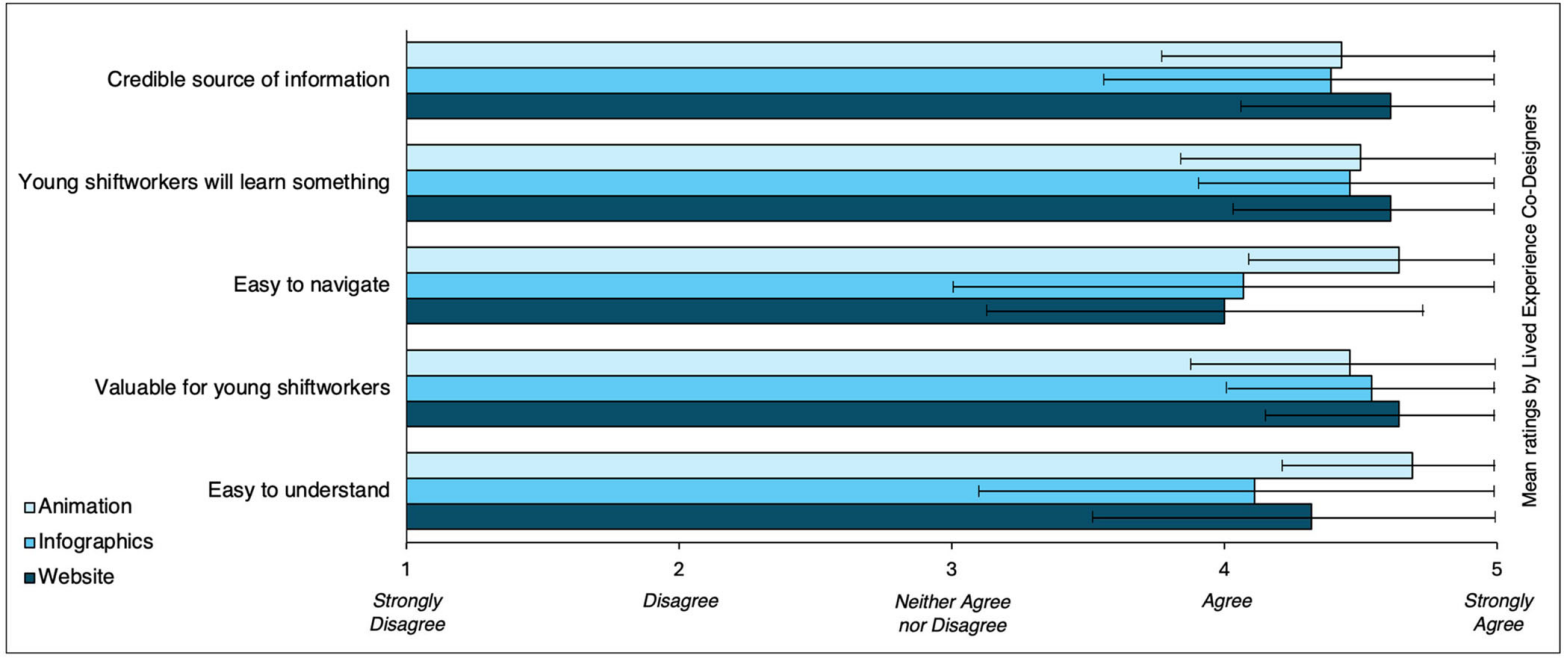
*Healthy from the Start* lived experience co‐designer feedback on resource utility. Error bars represent standard deviation (SD).

In response to Likert‐type scale rating questions on credibility, ease of use, ease of understanding, value and information quality across the suite of resources, the vast majority (91.7%) of responses were *agree* or *strongly agree* for each statement, as illustrated in Figure 3.

### 3.3 Research Question 3: Co‐Design Evaluation Outcomes

**Figure 4:**
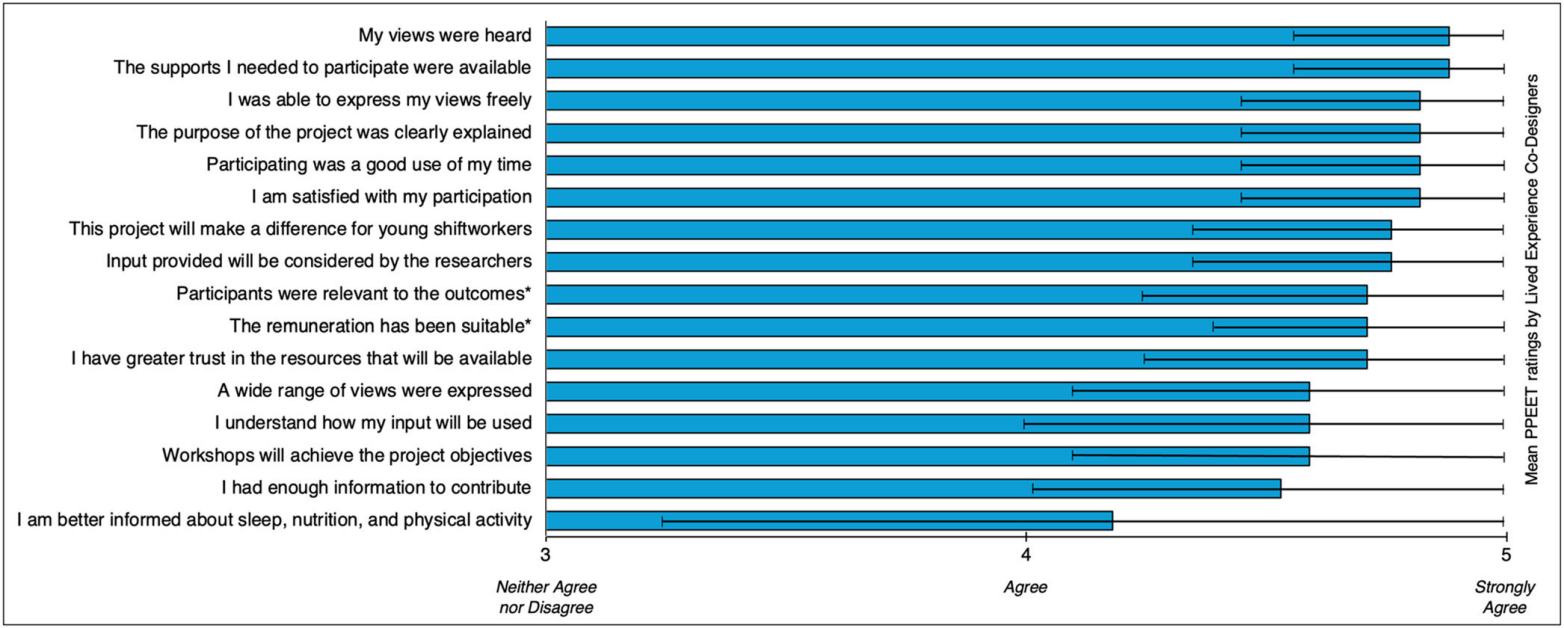
*Healthy from the Start* lived experience co‐design PPEET responses. Error bars represent standard deviation (SD). *denotes additional items added by the research team. PPEET = Public and Patient Engagement Evaluation Tool.

Of the 29 lived experience co‐designers who were eligible (i.e., those that completed both Co‐Design and Regroup workshops), 26 (89.7%) participated in Co‐Design Evaluation (i.e., completed the evaluation questionnaire after participating in the Regroup Workshops), with results reported in Figure 4.

The mean response across all statements was 4.7 (±0.2) on a 5‐point Likert‐type scale, with a rating of 4 indicating a response of *agree* and 5 indicating *strongly agree*. In response to how much of their input they felt had been captured and incorporated into the development of resources (out of 100), the mean rating across all lived experience co‐designers was 88.1% (±11.8%; range 50%–100%).

## 4 Discussion

This project involved the design, and novel implementation and evaluation of, a participatory, co‐design approach for developing sleep, nutrition and physical activity resources for young shiftworkers. In achieving this, lived experience co‐designers (i.e., those with lived experience of young shiftworker health and wellbeing separate to the academic experts) were engaged, not just for their lived experience input, but as true citizen scientists, contributing to the collection, interpretation, translation and dissemination of data [47]. A series of online workshops and questionnaires were used to collect mixed methods data from 48 co‐designers, including 36 lived experience co‐designers (young, experienced and previous shiftworkers, workplace health and safety experts and science communications specialists), and 12 academic experts. This novel approach addresses a significant gap in the literature where, to date, no such methodology has been utilised to develop health and/or wellbeing resources for shiftworkers.

Lived experience co‐designers provided positive feedback on the suite of resources developed, with the vast majority agreeing or strongly agreeing that these resources were a credible source of health information, easy to use and understand and would provide useful information for young shiftworkers. Further, lived experience co‐designers positively evaluated the co‐design approach used, reporting that their contributions were valued and clearly applied to the development of resources. For example, co‐designers reported that, on average, the vast majority (88.1%) of their contributions were not only captured but incorporated by the research team. While these outcomes reflect positively on the co‐design approach used and the resulting resources developed, it is challenging to situate them within the literature for several reasons. First, while participatory co‐design approaches are being used with increasing frequency, including in the development of health resources and interventions [28], it is acknowledged that such approaches do not routinely embed evaluative activities [48]. In the few instances when co‐designer feedback is sought, it usually relates to the project outcomes (i.e., the resources or interventions developed) or the co‐design process used, but rarely both [48]. Instead, the bulk of evidence in favour of co‐designed outputs is usually sought later from end‐users alone (e.g., healthcare service systems, clinicians, patients not involved in the project), rather than those who contributed to their development. As such, it is difficult to compare the positive feedback received from lived experience co‐designers in this project with existing literature. Second, co‐design has been rarely used in a shiftwork context. Shiftwork‐related research using a co‐design approach has largely focused on occupational elements (e.g., rostering practices) with minimal attention paid to shiftworker health and wellbeing [35, 36]. As such, these factors make it challenging to determine whether the positive reflections of lived experience co‐designers in this project are consistent with previous outcomes, and add weight to the argument for future shiftwork‐related projects to embed co‐design and participatory elements, particularly given their positive reflections of involvement in, and the outcomes from, the present study.

Despite the novel nature of the project, and the associated challenges in situating outcomes in the broader evidence base, it is nonetheless recommended that the shiftwork field consider the utility of participatory, co‐design approaches when developing health and wellbeing resources. For some time, it has been recommended that resources or health information aimed at shiftworkers be tailored to meet their unique circumstances, particularly in relation to individual and workplace health and wellbeing interventions [49, 50, 51]. To achieve high‐quality tailoring of resources that are fit for purpose, it is necessary to go beyond the replication of generic health information. This need not be viewed as contrary to traditional scientific paradigms, which centre academic expertise, but instead, as complementary, as it allows for the enrichment of scientific knowledge with lived experience insights [28, 52]. This approach is continuing to gain traction, particularly in healthcare‐related investigations [29], and it can be assumed that this mounting popularity is directly related to the superior engagement that is seen with the resultant co‐designed interventions amongst end‐users, when compared to those developed through more traditional scientific approaches [29]. As discussed, these positive outcomes in relation to engagement would be enriched by understanding feedback from co‐designers themselves. The evaluation of the approach used in this project, as well as feedback on the resulting resources, aimed to bridge this gap by encompassing both shiftwork and health behaviour focuses, providing early evidence for this novel approach being both feasible in achieving desired outcomes, as well as positive in the eyes of those with lived experience.

Beyond the positive evaluative outcomes, the participatory, co‐design approach proved feasible for developing sleep, nutrition and physical activity resources for young shiftworkers. The insights from both lived experience co‐designers and academic experts were readily translated into a suite of resources that are situated within the evidence base. The resources include a series of pictorial infographics, an animated video and a website housing extensive information on sleep, nutrition and physical activity. While lived experience co‐designers provided positive feedback on the resources, future investigations will be required to evaluate their efficacy, in terms of improving young shiftworkers’ knowledge of, and engagement with sleep, nutrition and physical activity health behaviours. It is recommended that several considerations inform such evaluations. First, validated measures should be used to explore changes in self‐reported health behaviour before and after resource implementation. This may include tools, such as the Pittsburgh Sleep Quality Index, to measure changes in sleep outcomes [53], the International Physical Activity Questionnaire, to measure changes in physical activity engagement [54] and food frequency questionnaires [55], to understand nutrition‐related outcomes. In addition to validated tools, measures examining theoretical elements that may underpin behaviour change (e.g., Theory of Planned Behaviour) should also be employed, to provide a nuanced understanding of changes in health behaviour, or lack thereof, in response to resource implementation [56, 57]. Finally, it would be beneficial to understand the level of knowledge change that may be occurring alongside health behaviour changes. Given the benefit of health education as a form of early intervention from a public health perspective [22, 25], identifying changes in young shiftworker knowledge before and after resource implementation will assist in understanding how knowledge change aligns with behaviour change, and areas that may require updated or additional information for young shiftworkers. This may be achieved by administering self‐assessment questions to young shiftworkers, to quantify their knowledge of sleep, nutrition and physical activity before and after resource implementation.

### 4.1 Strengths and Limitations

This project benefited from relatively high levels of participant retention throughout—the majority (86%) of lived experience co‐designers who enroled in the project participated in workshops, and of these, the majority completed process evaluation (82%) and resource evaluation (90%) activities. All academic experts remained engaged with the project throughout. While retention of lived experience co‐designers could be attributed, in part, to the remuneration that was offered, this is balanced by the intensive nature of involvement over a 6‐month period, as well as the relative pay scales of many shiftwork industries. As such, it is a strength of this project, and a benefit for the resulting resources, that lived experience co‐designers were able to maintain engagement throughout.

Lived experience co‐designers spanned a wide range of industries, which allowed for diverse input regarding the sleep, nutrition and physical activity challenges that young shiftworkers may face. While this is beneficial for the outputs of this project, it is worth noting that only a certain degree of diversity can be accomplished with a cohort of several dozen individuals. For example, certain industries were represented more than others; half of all lived experience co‐designers had experience in Healthcare and Social Assistance. While not all of these would have had clinical backgrounds (i.e., a clinical role in the healthcare system), it is worth considering whether these lived experience co‐designers had more highly developed knowledge of sleep, nutrition and physical activity as a result of this industry engagement and, therefore, applied this knowledge in the development of the resources (rather than knowledge gained from their lived experience of shiftwork itself). Of note, however, Healthcare and Social Assistance employs the highest number of shiftworkers in Australia (the population from which participants were drawn) [58]. While this may be representative of the broader community, it could also indicate an overrepresentation of this industry. Regardless, other industries (e.g., Mining, Public Administration and Safety) were represented, adding strength to the outcomes given the diversity of experiences across industries, and the infrequency with which multiple shiftwork industries are represented in a single investigation [59].

Co‐design projects necessitate restricting the number of participants to ensure meaningful engagement [43]. That is, the number of lived experience co‐designers that are recruited cannot exceed the resources available to facilitate their engagement. Case studies have demonstrated that online workshops, as used in this project, allow for purposeful contributions when participant numbers are restricted to 12 or fewer individuals (not including the facilitator/s) [43]. As such, 30–40 lived experience co‐designers were recruited and then split across three workshops. With natural attrition, it was assumed this would equate to 8–10 participants per workshop. While this did eventuate and, therefore, align with best‐practice approaches for conducting online workshops [43], this also limits the diversity of experiences and opinions to that of a few dozen individuals. To mitigate this, a Validation Workshop was held to seek the input of additional young shiftworkers, providing further insights beyond those gathered during the co‐design phases. Positively, this validation process was largely confirmatory of the data collected from earlier phases (i.e., aligned with the insights shared in earlier workshops).

While the inclusion of a Validation Workshop was beneficial in terms of corroborating the data collected in previous phases, it also served to strengthen the overall approach. As described by Slattery et al. [28] in their review of co‐design approaches in healthcare research, such mechanisms are not always included in co‐design projects. Instead, projects will traditionally involve participatory activities at either end—data collection and design, or data validation and dissemination—but rarely both [28]. As such, being able to implement this thorough approach contributes to the strength of the methodology and the application and utility of resulting outcomes. Beyond this, it is important to consider how the resulting resources will be implemented and evaluated, and the methods by which more diverse input can be sought. It should be noted that, despite achieving a degree of diversity in professional experience, other aspects of diversity (e.g., culture, geography, disability) were not captured across co‐designers. This is an important consideration for future investigations, given the implications such dimensions can have for health access, equity and literacy [60]. However, repeating the same participatory, co‐design approach risks altering the outputs beyond those that were created here. Instead, a pre‐post evaluation amongst a ‘generalist’ group of end‐users (young shiftworkers from a range of industries), which incorporates both qualitative and quantitative measures as described earlier, could provide rich insights that would allow for further tailoring or enhancement of the resources. In addition, the resources could be implemented with specific groups (e.g., regional and remote shiftworkers, shiftworkers from culturally and linguistically diverse backgrounds, shiftworkers living with disability) to explore their translation, utility and impact within these cohorts.

## 5 Conclusion

The implementation and evaluation of a novel approach to developing health and wellbeing resources for shiftworkers, using participatory, co‐design methods, has demonstrated both feasibility and positive evaluation amongst lived experience co‐designers. While the resulting resources on sleep, nutrition and physical activity require further investigation in terms of impacting knowledge and/or behaviour change amongst end‐users, early findings demonstrate that a participatory, co‐design approach is a valuable, viable tool at the disposal of researchers in the shiftwork health and wellbeing field.

## Author Contributions

**Alexandra E. Shriane:** conceptualisation, investigation, writing–original draft, methodology, validation, visualisation, writing–review and editing, formal analysis, project administration, data curation. **Sally A. Ferguson:** conceptualisation, investigation, funding acquisition, methodology, writing–review and editing, project administration, supervision, resources. **Gabrielle Rigney:** conceptualisation, investigation, funding acquisition, methodology, writing–review and editing, project administration, supervision. **Charlotte C. Gupta:** conceptualisation, investigation, funding acquisition, methodology, validation, writing–review and editing, formal analysis, project administration, data curation, resources. **Tracy Kolbe‐Alexander:** conceptualisation, methodology, writing–review and editing. **Madeline Sprajcer:** conceptualisation, methodology, writing–review and editing, funding acquisition. **Cassie Hilditch:** conceptualisation, funding acquisition, methodology, writing–review and editing. **Robert Stanton:** conceptualisation, funding acquisition, methodology, writing–review and editing. **Matthew J. W. Thomas:** conceptualisation, funding acquisition, methodology, writing–review and editing. **Jessica L. Paterson:** conceptualisation, funding acquisition, methodology, writing–review and editing. **Jamie Marino:** investigation, validation, data curation. **Grace E. Vincent:** conceptualisation, investigation, funding acquisition, methodology, validation, writing–review and editing, project administration, data curation, supervision, resources, formal analysis.

## Ethics Statement

Ethics approval was provided by the Human Research Ethics Committee at Central Queensland University, Australia, before commencing participant recruitment (Approval No. 24460).

## Conflicts of Interest

The authors declare no conflicts of interest.

## Supporting information

Supporting information.

## Data Availability

Deidentified data that supports the outcomes in this manuscript is available upon reasonable request from the corresponding author.
