# Supplementary material for: Healthy from the Start: Co‐Designing Sleep, Nutrition and Physical Activity Resources for Young Shiftworkers—Novel Implementation and Evaluation
Source: Health Expect. 2024 Oct 15;27(5):e70063. doi: 10.1111/hex.70063 (PMC11474620; doi:10.1111/hex.70063)
Supplement: Supplementary file 1 — Supporting information. [file HEX-27-e70063-s001.docx]

**Supplementary Material A. Co-Design Workshop Discussion Guide**

| **Topic** | **Primary Guiding Question** | **Additional Discussion Points** |
| --- | --- | --- |
| Sleep | What do we need to tell young shiftworkers about their sleep? | What do young shiftworkers already know about sleep?  How would this information differ from the general population?  How would this information differ from an older/more experienced shiftworker?  What information is general and what needs to be tailored to certain industries? |
| Nutrition | What do we need to tell young shiftworkers about their nutrition? | What do young shiftworkers already know about nutrition?  How would this information differ from the general population?  How would this information differ from an older/more experienced shiftworker?  What information is general and what needs to be tailored to certain industries? |
| Physical Activity | What do we need to tell young shiftworkers about their physical activity? | What do young shiftworkers already know about physical activity?  How would this information differ from the general population?  How would this information differ from an older/more experienced shiftworker?  What information is general and what needs to be tailored to certain industries? |
| Communication | How do we communicate this information to young shiftworkers? | What resources do young shiftworkers want or need?  Where are young shiftworkers getting their health and wellbeing information?  What should we avoid when communicating this information? |

**Supplementary Material B. Regroup Workshop Presentation & Discussion Guide**

| **Topic** | **Themes for Discussion and Feedback*** | Codes & draft copy for each topic presented for discussion and feedback* |
| --- | --- | --- |
| Sleep | - Sleep science basics - Impacts of poor sleep - Daily habits impacting sleep - Strategies and actions - Recommendations for workplaces |  |
| Nutrition | - Nutrition basics - Impacts of poor nutrition - Nutrition for shiftworkers - Strategies and actions - Recommendations for workplaces |  |
| Physical Activity | - Physical activity basics - Impacts of insufficient activity - Physical activity for shiftworkers - Strategies and actions - Recommendations for workplaces |  |
| Communication | - Digital resources - In-person resources - Framing and context | Example suite of resources presented for discussion and feedback* |

**Detailed description of themes, codes, and resultant resources, including approach to qualitative data analysis, to be provided in forthcoming publications.*
